# Primary tumor standardized uptake value (SUVmax) measured on ^18^F-FDG PET/CT and mixed NSCLC components predict survival in surgical-resected combined small-cell lung cancer

**DOI:** 10.1007/s00432-020-03240-8

**Published:** 2020-06-03

**Authors:** Zhenzhen Hui, Feng Wei, Hongliang Ren, Wengui Xu, Xiubao Ren

**Affiliations:** 1 Department of Biotherapy, Tianjin Medical University Cancer Institute and Hospital, Tianjin, 300060 People’s Republic of China; 2 Department of Immunology, Tianjin Medical University Cancer Institute and Hospital, Tianjin, 300060 People’s Republic of China; 3 National Clinical Research Center for Cancer, Tianjin, 300060 People’s Republic of China; 4 Key Laboratory of Cancer Prevention and Therapy, Tianjin, 300060 People’s Republic of China; 5 Tianjin’s Clinical Research Center for Cancer, Tianjin, 300060 People’s Republic of China; 6 Key Laboratory of Cancer Immunology and Biotherapy, Tianjin, 300060 People’s Republic of China; 7 Department of Molecular Imaging and Nuclear Medicine, Tianjin Medical University Cancer Institute and Hospital, Tianjin, 300060 People’s Republic of China; 8 Department of Radiology, Tangshan People’s Hospital, Tangshan, 063001 Hebei Province People’s Republic of China

**Keywords:** ^18^F-FDG PET/CT, c-SCLC, TNM stage, SUV_max_, SCC, PFS, Overall survival

## Abstract

**Purpose:**

The combined small-cell lung cancer (c-SCLC) is rare and has unique clinicopathological futures. The aim of this study is to investigate ^18^F-FDG PET/CT parameters and clinicopathological factors that influence the prognosis of c-SCLC.

**Methods:**

Between November 2005 and October 2014, surgical-resected tumor samples from c-SCLC patients who received preoperative ^18^F-FDG PET/CT examination were retrospectively reviewed. The maximum standardized uptake value (SUV_max_), metabolic tumor volume (MTV) and total lesion glycolysis (TLG) were used to evaluate metabolic parameters in primary tumors. The survivals were evaluated with the Kaplan–Meier method. Univariate and multivariate analyses were used to evaluate potential prognostic factors.

**Results:**

Thirty-one patients were enrolled, with a median age of 62 (range: 35 − 79) years. The most common mixed component was squamous cell carcinoma (SCC, *n* = 12), followed by large-cell carcinoma (LCC, n = 7), adenocarcinoma (AC, *n* = 6), spindle cell carcinoma (*n* = 4), adenosquamous carcinoma (*n* = 1) and atypical carcinoid (*n* = 1). The median follow-up period was 53.0 (11.0–142.0) months; the 5-year overall survival (OS) and progression-free survival(PFS) rate were 48.4% and 35.5%, respectively. Univariate survival analysis showed that gender, smoking history, tumor location were associated with PFS (*P* = 0.036, *P* = 0.043, *P* = 0.048), SUVmax and TNM stage were closely related to PFS in both Mixed SCC and non-SCC component groups (*P* = 0.007, *P* = 0.048). SUV_max_, smoking history, tumor size and mixed SCC component were influencing factors of OS in patients (*P* = 0.040, *P* = 0.041, *P* = 0.046, *P* = 0.029). Multivariate survival analysis confirmed that TNM stage (HR = 2.885, 95%CI: 1.323–6.289, *P* = 0.008) was the most significantly influential factor for PFS. High SUV_max_ value (HR = 9.338, 95%CI: 2.426–35.938, *P* = 0.001) and mixed SCC component (HR = 0.155, 95%CI: 0.045–0.530, *P* = 0.003) were poor predictors for OS.

**Conclusion:**

Surgical-resected c-SCLCs have a relatively good prognosis. TNM stage is the most significant factor influencing disease progression in surgical-resected c-SCLCs. SUVmax and mixed NSCLC components within c-SCLCs had a considerable influence on the survival. Both high SUVmax and mixed SCC component are poor predictors for patients with c-SCLCs.

## Introduction

Combined small-cell lung carcinoma (c-SCLC) is defined as small-cell lung cancer (SCLC) combined with an additional component that consists of any of the histological types of non-small-cell lung cancer (NSCLC), including adenocarcinoma (AC), squamous cell carcinoma (SCC), large-cell carcinoma (LCC), or spindle cell, carcinoid and other rare types (Travis 2014). C-SCLC is comparatively uncommon and accounts for only 1−3% of all SCLCs (Moon et al. 2019).

^18^F-fluorodeoxyglucose-positron emission tomography/computed tomography (^18^F-FDG PET/CT) imaging using the tracer ^18^F-FDG has emerged as an essential imaging tool for diagnosis and staging of lung cancer. The National Comprehensive Cancer Network guidelines have recommended the application of ^18^F-FDG PET/CT for SCLC patients (Johnson 2001). SUVmax measured on ^18^F-FDG PET/CT is used to quantify FDG uptake of tumor cells; the degree of tumor uptake of ^18^F-FDG on PET/CT is shown to be an valuable prognostic gauge in malignant tumors (Bai et al. 2017; Hsieh et al. 2018; Hsu et al. 2016; Kwon et al. 2016; Lee et al. 2015, 2018; Park et al. 2014, 2016; Zhu et al. 2018). While, volumetric parameters such as metabolic tumor volume (MTV) and total lesion glycolysis (TLG) are investigated for independent prognostic parameters in NSCLC and some other cancers (Albano et al. 2018; Burger et al. 2016; Hasbek et al. 2019; Lemarignier et al. 2017; Tsujikawa et al. 2017). ^18^F-FDG PET/CT is the main imaging tool for initial staging and influences patient management and early assessment of tumor response (Kim et al. 2018; Zer et al. 2016). With the development of ^18^F-FDG PET/CT technology, lung cancers are diagnosed earlier and more SCLC patients undergo surgery and have pathological examinations, which have led to more c-SCLC diagnoses recently (Qin and Lu 2018; Zhang et al. 2017). However, influence of primary tumor metabolic parameters and mixed NSCLC components on survival of c-SCLC, and whether they are associated with prognosis are unclear.

The present study was performed to examine whether preoperative metabolic parameters of primary tumors measured on ^18^F-FDG PET/CT and mixed NSCLC components are correlated with overall survival in surgical-resected c-SCLC.

## Materials and methods

### Patients and diagnosis

The Ethics Committee of Tianjin medical university cancer institute and hospital (TMUCIH) approved this study, which was carried out in accordance with the Declaration of Helsinki. The requirement for informed consent was waived as the study was retrospective.

A retrospective review of postoperative lung cancer patients who had ^18^F-FDG PET/CT examination before surgery in TMUCIH between November 2005 and October 2014 was conducted. During this period, 1035 patients underwent ^18^F-FDG PET/CT examinations and surgical resection of primary lung cancers at the Department of Thoracic Surgery of our institution. Thirty-seven (3.6%) patients were diagnosed with c-SCLC, six patients with incomplete clinical and follow-up data were excluded. Thirty-one (3.0%) consecutive patients with pathologically confirmed c-SCLC were retrospectively reviewed, based on the diagnostic criteria proposed by the 2015 edition of the WHO classification system. Each surgically resected tumor was systematically sampled according to standard principles. Paraffin-embedded tumor specimens, which included the widest cross sections, were reassessed by two senior clinical pathologist. Immunohistochemistry staining of surgically resected c-SCLC was used to for the modification of the classification of SCLC and non-SCLC components within c-SCLC.

### Neoadjuvant and adjuvant treatment

Two cycles of neoadjuvant chemotherapy (EP regimen) was performed in 3 patients who underwent pneumonectomy. Twenty-five patients accepted adjuvant chemotherapy with EP regimen or EP combined with TP, GP or AP regimen, and one patient with EGFR mutation in mixed adenocarcinoma component was given gefitinib as adjuvant therapy. Five patients with stage I and II A did not accept adjuvant chemotherapy treatment. The EP regimen was etoposide 100 mg/m^2^ (days 1–3) and cisplatin or carboplatin (cisplatin 75 mg/m^2^, carboplatin AUC = 5–6; day 1). The TP regimen was paclitaxel 135–175 mg/m^2^ (days 1) and cisplatin or carboplatin (cisplatin 75 mg/m^2^, carboplatin AUC = 5–6; day 1). The GP regimen was gemcitabine 1000–1250 mg/m^2^ (days 1 and 8) and cisplatin or carboplatin (cisplatin 75 mg/m^2^, carboplatin AUC = 5–6; day 1). The AP regimen was pemetrexed 500 mg/m^2^ (days 1) and cisplatin or carboplatin (cisplatin 75 mg/m^2^, carboplatin AUC = 5–6; day 1). Chemotherapy was administered at 3-week intervals for total 4–6 cycles. Local radiotherapy and prophylactic brain irradiation (PCI) were given in 11 patients. 3D conformal radiotherapy or intensity-modulated radiotherapy (PTV, 54 Gy/30f) was administered concurrent or followed chemotherapy. The radiotherapy fields covered primary lesions, hilar and ipsilateral mediastinal lymph nodes. Finally, PCI (25 Gy/10f) was performed.

### ^18^F-FDG PET/CT imaging and interpretation

In our study, all patients (*n* = 31) underwent preoperative ^18^F-FDG PET/CT examination to confirm clinical stage and exclude distant metastasis. PET/CT scans were performed using a GE Discovery Elite PET/CT scanner (GE Medical Systems, Waukesha, WI, USA). All patients were requested to fast for at least 6 h prior to the ^18^F-FDG PET/CT scan. Serum glucose levels were measured before the ^18^F-FDG injection; no patient had a glucose level that exceeded 6.8 mmol/L. FDG was administered intravenously at a dose of 4.2 MBq ^18^F-FDG/kg body weight. After an hour, a spiral CT scan with ~ 25 effective mAs, 130 kVp, and a 5-mm slice thickness was taken, followed by a PET emission scan from the distal femur to the top of the skull (Yu et al. 2017a, b).

Two board-certified nuclear medicine physicians reviewed the PET/CT images side by side and calculated the area SUVmax, MTV, and TLG using line attenuation correction and iterative reconstruction of the image in the manually constructed radionuclide focal volume of interest (VOI). SUVmax was defined as the highest pixel value. The tumor size was expressed by the maximum diameter measured on the lung window in CT.

### Follow-up

Patients were followed-up every 3 months for the first year and then every 3–6 months thereafter. Methods to obtain follow-up information include: communication with physicians, looking up to inpatient or outpatient records, death certificates, and communication with patient or patient’s family. Progression-free survival (PFS) was defined as the interval from the date of resection to the date of proven detection of local recurrence or metastasis. The duration of overall survival (OS) was defined as the interval between the day of surgery and the date of death by any cause or the last follow-up date. The primary end-point of the study was OS.

### Statistical analysis

Pearson correlation analysis and Spearman rank correlation analysis were used, respectively, according to whether the variables were normally distributed or not. Kaplan–Meier analysis was used for univariate survival analysis and compared using the log-rank test. Cox risk regression model was used for multivariate analysis affecting prognosis. Significant predictors of univariate analysis (*P* < 0.05) supported by clinical evidence were included in the Cox’s multivariate analysis. Backward stepwise (Likelihood Ratio) was used to estimate the association between the predictors and outcomes, using hazard ratio (HR) and its 95% confidence interval (95% CI) as the indicators. *P* value < 0.05 (two sided) was considered statistically significant. Statistical analyses were performed using SPSS software (version 23.0; IBM-SPSS, Inc., Chicago, IL, USA).

## Results

### Patient characteristics

A total of 31 patients diagnosed with c-SCLC were included in the present study. Patient clinical characteristics are listed in Table 2. The median age was 62 (range: 35 − 79) years. Most patients had a history of smoking (*n* = 22, 71.0%) and patients were overwhelmingly male (5.2:1). C-SCLC developed predominantly in peripheral sites (*n* = 23, 74.2%). Of these patients, a lobectomy was performed in 21 patients, while bilobectomy was performed in 3, pneumonectomy in 3, and 4 patients underwent a wedge resection. Radical mediastinal lymph node dissection was performed in 29 patients. Two IA patients with wedge resection did not receive lymph node dissection. In our study, squamous cell carcinoma (SCC, *n* = 12) was the most common mixed component (Men et al. 2016), followed by large-cell carcinoma (LCC, *n* = 7), adenocarcinoma (AC, *n* = 6), spindle cell carcinoma (*n* = 4), adenosquamous carcinoma (n = 1) and atypical carcinoid (*n* = 1). The final pathologic lung cancer stages in the patients were as follows: stage IA in 8 patients, IB in 5, IIA in 8, IIIA in 9 and IIIB in 1.

### The relationship between tumor metabolic status and clinicopathological characteristics

Pearson or Spearman rank correlation analysis was used to analyze the relationship between primary tumor metabolic parameters (SUV_max_, MTV, TLG) and clinicopathological features, including gender, age, smoking history, tumor location, tumor size, Lymph node metastasis, mixed NSCLC components, TNM stage, SCC, NSE, CEA, white blood cell (WBC) count, neutrophil, lymphocyte, neutrophil–lymphocyte ratio (NLR), platelet–lymphocyte ratio (PLR) and hemoglobin (HGB). Primary tumor SUVmax measured on ^18^F-FDG PET/CT has no significant correlation with clinicopathological factors. Both MTV and TLG were significant correlated with tumor size, WBC and lymphocyte count (MTV: *P* < 0.001, *P* = 0.023, *P* < 0.001; TLG: *P* < 0.001, *P* = 0.009, *P* < 0.001, Table 1).

**Table 1:** The relationship between primary tumor 18F-FDG PET/CT metabolic parameters and clinicopathological characteristics

Variables	SUVmax		MTV (cm^3^)		TLG (g/ml × cm^3^)	
	*r*	*p*	*r*	*p*	*r*	*p*
Gender	− 0.064	0.733	− 0.161	0.433	− 0.297	0.141
Age△	0.075	0.689	− 0.161	0.431	0.100	0.629
Smoking index △	0.087	0.640	− 0.194	0.341	− 0.231	0.255
Tumor location	− 0.169	0.363	− 0.111	0.589	− 0.189	0.355
Tumor size	0.150	0.421	0.754	< 0.001***	0.717	< 0.001***
LN metastasis	0.037	0.845	0.072	0.727	0.082	0.689
Mixed component	0.192	0.319	0.210	0.326	0.227	0.285
TNM stage	0.095	0.610	0.284	0.160	0.251	0.217
SCC △	0.076	0.725	0.223	0.345	0.254	0.280
NSE △	− 0.005	0.982	− 0.217	0.333	− 0.200	0.372
CEA △	− 0.244	0.220	− 0.090	0.689	− 0.119	0.598
WBC△	0.020	0.915	0.452	0.023*	0.508	0.009**
Neutrophil △	− 0.039	0.838	0.197	0.345	0.256	0.216
Lymphocyte △	0.146	0.442	0.714	< 0.001***	0.720	< 0.001***
NLR △	− 0.182	0.335	− 0.181	0.387	− 0.133	0.525
PLR △	− 0.322	0.083	− 0.363	0.075	− 0.347	0.089
HGB △	− 0.223	0.237	0.024	0.908	− 0.007	0.974

Δ, Pearson correlation analysis; the others: Spearman rank correlation analysis

*SUV*_*max*_ maximum standardized uptake value; *MTV* metabolic tumor volume; *TLG* total lesion glycolysis; *LN* lymph node; *TNM* tumor–node–metastasis; *SCC* squamous cell carcinoma antigen; *NSE* neuron specific enolase; *CEA* carcinoembryonic antigen; *WBC* white blood cell; *NLR* neutrophil–lymphocyte ratio; *PLR* platelet–lymphocyte ratio; *HGB* hemoglobin

*p < 0.05; **p < 0.01; ***p < 0.001

### Survival analysis

**Fig. 1:**
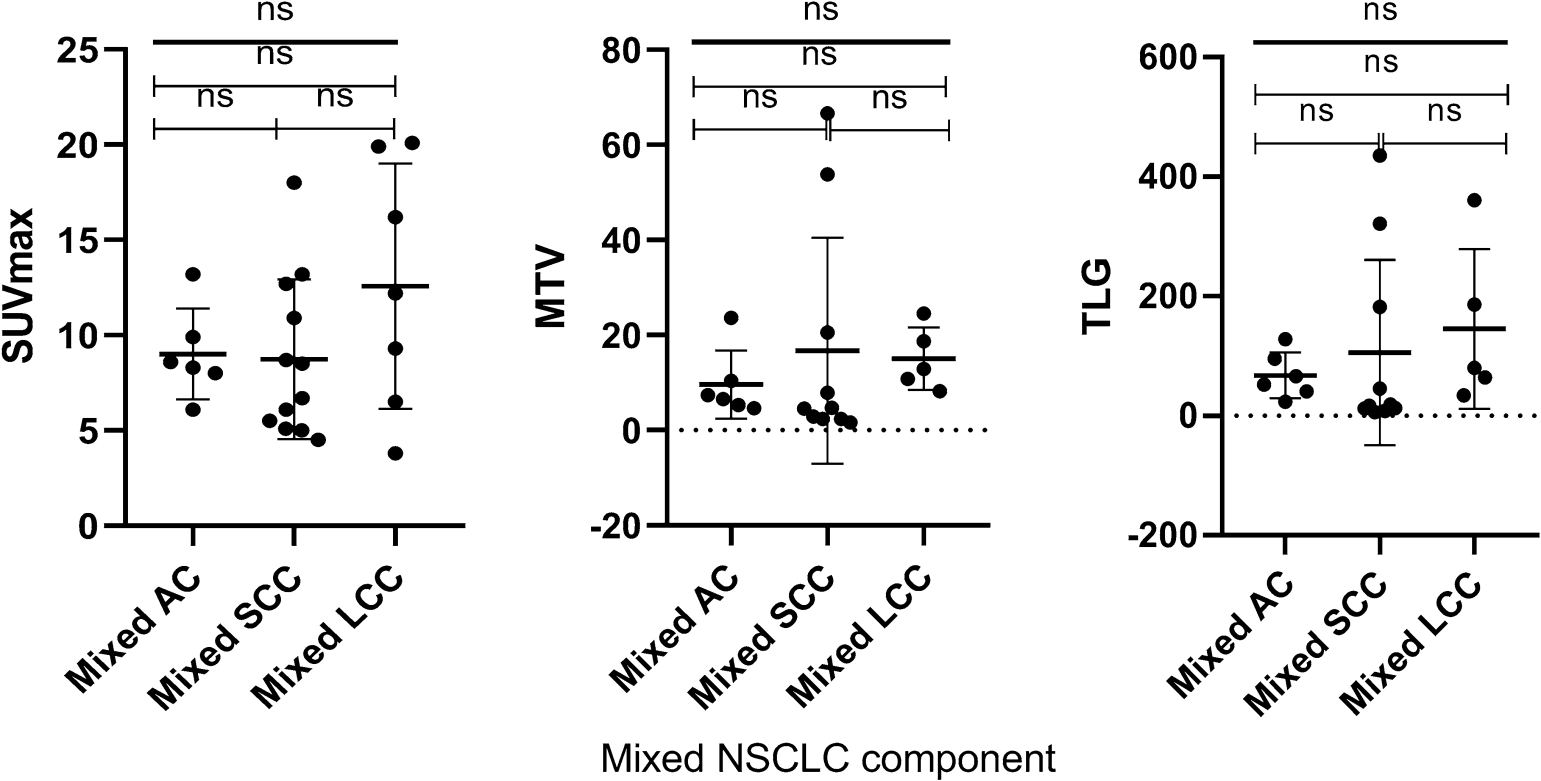
Primary tumor ^18^F-FDG PET/CT metabolic parameters distribution and inter-group comparison of common mixed NSCLC components

**Fig. 2:**
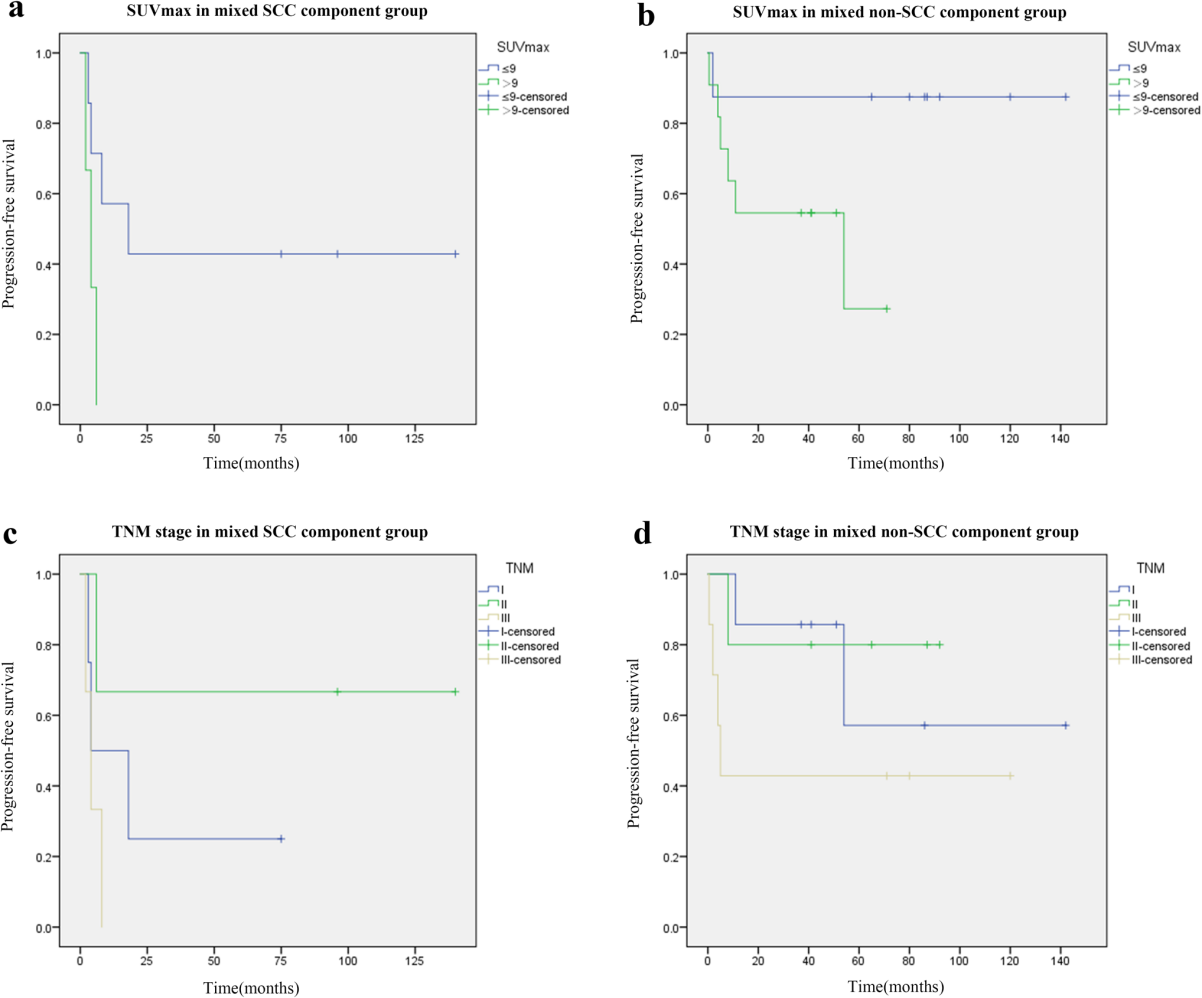
Progression-free survival curves for (**a**, **b**) SUVmax in mixed SCC and non-SCC component group (*P* = 0.007); **c**, **d** TNM stage in mixed SCC and non-SCC component group (*P* = 0.048)

**Fig. 3:**
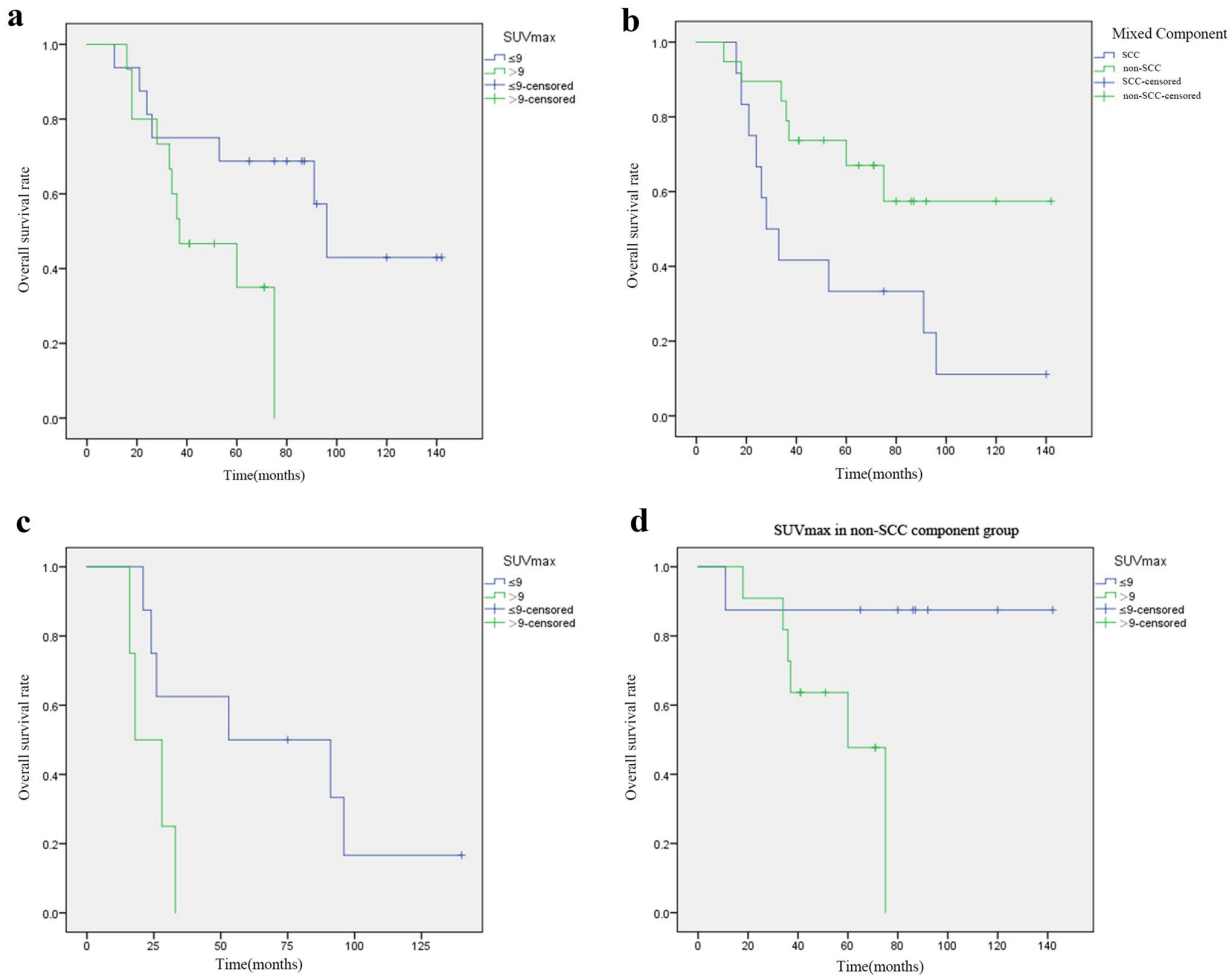
Overall survival curves for **a** SUV_max_ of primary tumor (*P* = 0.040); **b** SCC component vs non-SCC component group (*P* = 0.029); **c**, **d** SUV_max_ in mixed SCC and non-SCC component group (*P* = 0.004)

There was no treatment-related death in this cohort and all deaths were due to the primary disease. The median follow-up period was 53.0 (11.0–142.0) months. At the end of follow-up, nine patients had local recurrence and seven developed distant metastasis to bone, brain and liver. The 3-year and 5-year overall survival(OS) rate was 67.7% and 48.4%, corresponding progression-free survival(PFS) rate were 51.6% and 35.5%, respectively. Enrolled patients had the minimum SUVmax value of 3.3 and the maximum SUVmax value of 20.1. The minimum value of MTV and TLG was 1.65 cm^3^ and 6.17 g/ml × cm^3^, maximum value was 2179.0 cm^3^ and 3919.3 g/ml × cm^3^, respectively. Tumor metabolic parameters distribution and inter-group comparison of common mixed NSCLC components are shown in Fig. 1 (One-way ANOVA, *P* > 0.05). There was no significant difference between the common mixed NSCLC component groups. We used RStudio (R version 3.6.1) to draw the time-dependence ROC curve and get the optimal cutoff value (SUV_max_ = 9.0, MTV = 10.35 cm^3^, TLG = 128.23 g/ml × cm^3^). Univariate survival analysis showed that gender, smoking history, tumor location were prognostic factors of PFS (*P* = 0.036, *P* = 0.043, *P* = 0.048, Table 2). Male, non-smoking and peripheral c-SCLCs had a relatively longer PFS. SUVmax, mixed NSCLC component, tumor size, TNM stage and chemotherapy were associated with PFS, but not statistically significant (0.05 < *P* < 0.1, Table 2). We further analyzed the predictive value of these factors in the mixed SCC and non-SCC component groups separately and found that SUVmax and TNM stage were closely related to disease progression in both SCC and non-SCC component groups (*P* = 0.007, *P* = 0.048*,* Fig. 2). Kaplan–Meier survival analysis showed that SUVmax, smoking history, tumor size and mixed SCC component were influencing factors of OS in patients (*P* = 0.040, *P* = 0.041, *P* = 0.046, *P* = 0.029, Table 2). Moreover, stratified analysis showed that the SUVmax of mixed SCC group and non-SCC group were significantly correlated with OS (*P* = 0.004, Fig. 3).

**Table 2:** Patients characteristics and univariate analysis of c-SCLC (*N* = 31)

Characteristics	*n* (%)	PFS		OS	
		Median (95% CI)	*p* value	Median (95% CI)	*p* value
Gender			0.036*		0.249
Male	26 (83.9)	__a_		75.0 (34.6–115.4)	
Female	5 (16.1)	4.0 (0.0–8.3)		34.0 (21.1–46.9)	
Age(year)			0.681		0.455
≤ 62	15 (48.4)	18.0		91.0 (61.2–120.8)	
> 62	16 (51.6)	__a_		37.0 (0.0–77.3)	
Smoking history			0.043*		0.041*
Absence	9 (29.0)	__a_		__a_	
Presence	22 (71.0)	11.0 (0.0–24.5)		53.0 (18.0–88.0)	
Tumor location			0.048*		0.073
Central	8 (25.8)	__a_		26.0 (13.5–38.5)	
Peripheral	23 (74.2)	6.0 (2.3–9.7)		91.0 (50.3–131.7)	
SUV_max_			0.084		0.040*
≤ 9	16 (51.6)	__a_		96.0 (84.8–107.2)	
> 9	15 (48.4)	8.0 (0.0–17.2)		37.0 (10.7–63.3)	
MTV			0.272		0.347
≤ 10.35	16 (51.6)	18.0		75.0 (17.7–132.3)	
> 10.35	10 (32.3)	__a_		__a_	
TLG			0.552		0.793
≤ 128.23	20 (64.5)	__a_		91.0 (70.0–112.0)	
> 128.23	6 (19.4)	__a_		37.0	
Mixed component			0.072		0.083
SCC	12 (38.7)	6.0 (0.0–12.2)		28.0 (16.1–39.9)	
AC	6 (19.4)	__a_		__a_	
LCC	7 (22.6)	__a_		__a_	
Spindle cell	4 (12.9)	5.0 (0.0–13.8)		34.0 (9.5–58.5)	
Others	2 (6.5)				
Mixed Component			0.086		0.029*
SCC	12 (38.7)	6.0 (0.0–12.2)		28.0 (16.1–39.9)	
Non-SCC	19 (61.3)	__a_		100.0 (75.3–124.7)	
Tumor size			0.055		0.046*
≤ 3 cm	20 (64.5)	11.0 (0.0–23.8)		53.0 (0.4–105.6)	
> 3 cm	11 (35.5)	__a_		__a_	
TNM stage			0.068		0.365
I	13 (41.9)	54.0		60.0 (27.3–92.7)	
II	8 (25.8)			96.0 (65.0–127.0)	
III	10 (32.3)	4.0 (0.9–7.1)		33.0 (20.6–45.4)	
Radiotherapy			0.292		0.815
Yes	11 (35.5)	8.0 (0.0–60.9)		75.0 (20.1–129.9)	
No	20 (64.5)	__a_		91.0 (14.5–167.5)	
Chemotherapy			0.085		0.630
Yes	26 (83.9)	__a_		75.0 (35.0–115.0)	
No	5 (16.1)	__a_		__a_	
Surgical approach			0.659		0.700
Thoracotomy	24 (77.4)	11.0		75.0 (11.8–138.2)	
VATS	7 (22.6)	54.0		60.0 (45.6–74.4)	
Type of resection			0.496		0.501
Lobectomy	21 (67.7)	__a_		91.0 (26.9–155.1)	
Bilobectomy	3 (9.7)	__a_		__a_	
Pneumonectomy	3 (9.7)	8.0 (4.8–11.2)		75.0 (0–169.4)	
Wedge resection	4 (12.9)	2.0		28.0 (0–69.2)	
NSE			0.179		0.543
≤ 15 ug/L	17 (63.0)	__a_		60.0 (31.9–88.1)	
> 15 ug/L	10 (37.0)	6.0 (0.0–14.2)		34.0 (29.4–38.6)	
CEA			0.658		0.147
≤ 5 ug/L	22 (81.5)	18.0		75.0 (38.5–111.5)	
> 5 ug/L	5 (18.5)	54.0 (0.0–130.3)		36.0 (18.8–53.2)	
HGB			0.545		0.464
≤ 138.5	15(50.0)	__a_		96.0 (10.8–181.2)	
> 138.5	15 (50.0)	18.0 (0.0–79.3)		60.0 (24.0–96.0)	
NLR			0.951		0.684
≤ 2.24	15 (50.0)	54.0 (0.0–126.9)		75.0 (41.0–109.0)	
> 2.24	15 (50.0)	__a_		37.0 (0.0–92.0)	
PLR			0.504		0.604
≤ 121.9	15 (50.0)	54.0		60.0 (31.2–88.8)	
> 121.9	15 (50.0)	11.0		91.0 (0.0–183.1)	

*PFS* progression-free survival; *OS* overall survival; *CI* confidence interval; *SUV*_*max*_ maximum standardized uptake value; *MTV* metabolic tumor volume; *TLG* total lesion glycolysis; *SCC* squamous cell carcinoma; *AC* adenocarcinoma; *LCC* large-cell carcinoma; *TNM* tumor–node–metastasis; *VATS* video-assisted thoracoscopic surgery; *NSE* neuron specific enolase; *CEA* carcinoembryonic antigen; *HGB* hemoglobin; *NLR* neutrophil–lymphocyte ratio; *PLR* platelet–lymphocyte ratio

*Significantly different

−^a^ Median survival could not be estimated because more than half of the patients in the corresponding subgroup are alive

In Cox’s multivariate analysis, TNM stage (HR = 2.885, 95%CI: 1.323–6.289, *P* = 0.008) was the most significantly influential factor for PFS. High SUV_max_ value (HR = 9.338, 95%CI: 2.426–35.938, *P* = 0.001) and mixed SCC component (HR = 0.155, 95%CI: 0.045–0.530, *P* = 0.003) were poor prognostic factors for OS. The final analysis showed that besides TNM stage, SUV_max_ and mixed NSCLC components were important predictors of c-SCLC patients.

## Discussion

C-SCLC is a rare tumor with independent biological characteristics (Babakoohi et al. 2013; Qin and Lu 2018). Previous reports showed that up to 28% of SCLC patients who underwent surgical resection were c-SCLC (Nicholson et al. 2002). Fushimi et al. (1996) also reported that the frequency of c-SCLC in the primary sites was statistically higher in autopsy specimens (14.3%) than in biopsy or cytology specimens (8.6%). A retrospective study conducted by zhao et al. showed that 5.9% of surgically excised SCLC patients were c-SCLCs (Zhao et al. 2019). For patients diagnosed based on limited biopsy material, such as bronchial biopsy or needle aspiration, the possibility of detecting a combined histology is lower due to the limited amount of biopsy specimens. Our research subjects were all the pathological diagnosis after surgical resection, which ensured the accuracy and reliability of the diagnosis.

Conventionally, the treatment of c-SCLC refers to the guidelines for SCLC, and multimodality therapy is often recommended. Surgery plays an increasing role in limited-stage SCLCs, especially in c-SCLCs. A retrospective study conducted by Zhao et al. (2019) showed that 5-year survival rates of surgical-resected SCLC were 63.8%, 65.5%, and 34.9% for pathologic stages I, II and III, respectively, and suggested that surgery may also have potential benefit for stage II and some stage IIIA SCLC patients. In our study cohort, c-SCLCs are mainly peripheral located (74.2%) and earlier stage. All patients undergo radical resection, the most common mixed component is SCC, which is consistent with previous studies (Fraire et al. 1992; Hage et al. 1998; Men et al. 2016). In our study cohort, the 5-year overall survival and progression-free survival rate were 48.4% and 35.5%, suggesting that surgery is critical for c-SCLC because it not only provides an accurate diagnosis but also improves treatment outcomes (Stinchcombe 2017; Veronesi et al. 2015).

All our patients had a SUV_max_ value of > 2.5, the optimum cutoff value of SUV_max_, MTV and TLG was 9.0, 10.35 and 128.23, respectively. Pearson and spearman correlation analysis showed that MTV and TLG were significant correlated to tumor size, WBC and lymphocyte count. The WBC count before treatment was an indicator of systemic inflammation. We found that volumetric parameters MTV and TLG were closely related to hematological WBC count. A recent study conducted in 73 advanced HNSCC patients (Ohashi et al. 2020) proved that WBC count was significantly correlated with ^18^F-FDG PET/CT parameters, and speculated that tumor with upregulated aerobic glycolysis produce large amounts of lactic acid and cytokines and might mediate systemic inflammation via the lactic acid-induced IL-23/IL-17 pathway. Several studies also confirmed the relationship between PET-CT volumetric metabolic parameters and NLR/PLR in SCLC, NSCLC, cervical carcinoma and colorectal cancer (Du et al. 2019; McSorley et al. 2018; Mirili et al. 2019; Wang et al. 2020).

We included 31 surgical-resected c-SCLC patients with preoperative ^18^F-FDG PET/CT examination in our study and demonstrate that TNM stage was the most significantly influential factor for PFS, high SUVmax and mixed SCC component of the primary lesions were poor predictors of OS in c-SCLCs. A cohort study of 5002 patients (Nicholson et al. 2016) has confirmed the prognostic value of both clinical and pathologic TNM staging in SCLC patients with limited-stage disease. Several studies have reported that high SUV_max_ values in ^18^F-FDG PET/CT as a prognostic factor are associated with a poorer clinical outcome in patients with various malignancies, such as head and neck cancer, renal cell carcinoma, cervical cancer, gastric cancer and NSCLC (Bille et al. 2013; Brunette et al. 2018; Chon et al. 2019; Ha et al. 2017; Pankowska et al. 2019). Kwon et al. (2016) conducted a retrospective study and enrolled 59 limited-stage SCLC patients who underwent pretreatment ^18^F-FDG PET/CT and found that highest SUV_max_ is an independent prognostic factor for survival in limited-stage SCLC patients. Chang et al. (2019) analyzed the prognostic implication of ^18^F-FDG PET/CT in 30 LD-SCLC patients who underwent standard chemotherapy after radiotherapy and confirmed that SUV_max_ measured on pretreatment ^18^F-FDG PET/CT were independent and significant prognostic factors in LD-SCLC patients after chemoradiotherapy with curative intent. The percentile (%) change in SUV_max_ during and after treatment might be a better surrogate marker of clinical efficacy of chemotherapy compared to a single pre-treatment SUV_max_ value. A group of Korean investigators (Kim et al. 2018) compared ^18^F-FDG PET/CT parameters obtained from two consecutive PET/CT scans performed before and after treatment in 59 SCLC patients to predict prognosis. The results showed a significant reduction in SUV_max_ following treatment was an important independent prognostic factor for overall survival.

Mixed SCC component is another important prognostic indicator. Although most patients in the SCC component group were early-stage patients with lower SUVmax value, their prognosis was still poor, suggesting that mixed NSCLC components had independent and significant prognostic value for c-SCLC. Consistent with our study, Men et al. (2016) confirmed that the most common mixed component was SCC in 114 c-SCLCs, but survival analysis showed no significant difference between the SCC and non-SCC component group (*P* = 0.198), perhaps due to the different TNM stages of enrolled patients and only half of their patients had surgery. Small case series suggest that EGFR-TKI could also be used in c-SCLC with EGFR mutations (Okamoto et al. 2006; Tatematsu et al. 2008; Zakowski et al. 2006). EGFR mutations are more likely found in c-SCLC with adenocarcinoma component. In this study, there was one case of c-SCLC mixed with 60% adenocarcinoma accompanied by chest wall invasion and EGFR21 mutation. After surgery, gefitinib was given as adjuvant therapy, with a total survival of 18 months. Previous published studies were also scattered case reports, so it is difficult to accurately evaluate their efficacy because of data sparsity (Lu et al. 2012; Okamoto et al. 2006; Takagi et al. 2013; Zakowski et al. 2006).

There are some deficiencies in this study: first, it is a retrospective study; second, the sample size is small; third, the lack of uniform adjuvant treatment. In addition, because it was a single-center retrospective study, the results may be biased.

## Conclusion

We conducted a retrospective study of surgical-resected c-SCLC patients with preoperative ^18^F-FDG PET/CT examination. Primary tumor SUVmax measured on ^18^F-FDG PET/CT has no significant correlation with clinicopathological factors. Volumetric parameters MTV and TLG are significantly correlated with tumor size, WBC and lymphocyte count. TNM stage is the most significant factor influencing disease progression in surgical-resected c-SCLCs. Both high SUVmax value and mixed SCC component are poor prognostic factors in patients with c-SCLC. Surgical-resected c-SCLCs have a relatively good prognosis; multidisciplinary combination therapy is the main treatment mode for c-SCLC, especially for limited-stage disease. Due to the limitations of our research, these observations should be confirmed by further large-scale studies.
